# The Effect of Exploration on the Use of Producer-Scrounger Tactics

**DOI:** 10.1371/journal.pone.0049400

**Published:** 2012-11-21

**Authors:** Ralf H. J. M. Kurvers, Steven Hamblin, Luc-Alain Giraldeau

**Affiliations:** 1 Resource Ecology Group, Wageningen University, Wageningen, The Netherlands; 2 Department of Biology and Ecology of Fishes, Leibniz-Institute of Freshwater Ecology and Inland Fisheries, Berlin, Germany; 3 Department of Biological Sciences, Université du Québec à Montréal, Montreal, Canada; 4 School of Biotechnology and Biomolecular Sciences, University of New South Wales, Sydney, Australia; University of Bristol, United Kingdom

## Abstract

Individuals foraging in groups can use two different tactics for obtaining food resources. Individuals can either search for food sources themselves (producing) or they can join food discoveries of others (scrounging). In this study we use a genetic algorithm in a spatially explicit producer-scrounger game to explore how individuals compromise between exploration (an important axis of animal personality) and scrounging and how characteristics of the environment affect this compromise. Agents varied in exploration and scrounging and a genetic algorithm searched for the optimal combination of exploration and scrounging. The foraging environments featured different levels of patch richness, predation and patch density. Our simulations show that under conditions of low patch densities slow exploring scroungers were favored whereas high patch density favored fast exploring individuals that either produced (at low patch richness) or scrounged (at high patch richness). In high predation environments fast exploring individuals were selected for but only at low to intermediate patch densities. Predation did not affect scrounging behavior. We did not find a divergence of exploration ‘types’ within a given environment, but there was a general association between exploration and scrounging across different environments: high rates of scrounging were observed over nearly the full spectrum of exploration values, whereas high rates of producing were only observed at high exploration values, suggesting that cases in which slow explorers start producing should be rare. Our results indicate that the spatial arrangement of food resources can affect the optimal social attraction rules between agents, the optimality of foraging tactic and the interaction between both.

## Introduction

Individuals foraging in groups can use two different tactics to obtain food resources. Individuals can either search for food sources themselves (producing) or they can join food discoveries of others (scrounging). The first producer-scrounger models that were developed assumed that all individuals in the population were homogenous (a symmetric game) [1]–[3] providing insights into the equilibrium proportion of scroungers under different conditions. It is well-known that individuals in foraging groups are not always equal, and subsequent models investigated how differences between individuals (or within individuals over time) might affect producer-scrounger dynamics. Studies have investigated the effect of differences in dominance [4]–[6], search efficiency [6], vigilance level [7] and metabolic rate [8] on producing and scrounging pay-offs, providing insights in how differences between individuals affect producer-scrounger dynamics.

Recently, the field of animal personality has gained considerable attention. Animal personality describes the phenomenon that differences among individuals of the same species in behavioural and physiological traits are consistent over time and context and that different behavioural traits are correlated [9]–[12]. However, few attempts have been made to incorporate personality differences in producer-scrounger models, whereas experimental evidence is accumulating that personality traits can affect producer-scrounger dynamics [13]–[16]. Here we investigate how individuals compromise between scrounging behavior and exploration and how environmental differences affect this compromise. Exploration is an important axis of animal personality and has been documented in a wide variety of species [17]. Exploration is a prominent candidate to affect the optimal level of scrounging since exploration affects the tendency to move away from conspecifics and explore the environment. Several studies in group-living species show that there can be consistent differences in space use between individuals of the same species: at one extreme there are individuals that explore the environment and move far away from conspecifics, and at the other extreme are individuals that stay close to conspecifics and explore less [18]–[22]. These differences in spatial behavior are likely to affect the value of the different foraging tactics (i.e., producing or scrounging) since the value of foraging tactics depends on spatial proximity to conspecifics [23]–[25]. Here we use the terminology ‘slow’ and ‘fast’ explorers to describe the extreme ends of the exploration axis, this is in agreement with animal personality literature investigating exploration [26]–[28].

In order to understand how individuals compromise between exploration and scrounging tactic, we used a genetic algorithm approach [29] in a spatially explicit producer-scrounger model. Genetic algorithms mimic the action of natural selection to model population change over time [23], [30], [31], selecting the most successful individuals (i.e. those with the highest foraging rates) to reproduce in the next generation. We allowed agents in the simulation to vary in exploration, defined as their tendency to explore the environment by moving away from other conspecifics, and in scrounging probability. The genetic algorithm searched for the optimal combination of exploration and scrounger strategy use. To understand how individuals compromise exploration and scrounging tactic under different ecological conditions, we varied patch density, patch richness and predation. Of particular interest is an earlier study [23] investigating producer-scrounger roles and spatial position. This study found that groups consisting of producers and scroungers are more compact than groups of producers only and that scrounger are mainly found in central positions. Though illustrating that spatial dynamics and foraging tactic are linked the model [23] has some important assumptions: regardless of distance from a producing group mate scroungers arrive in one time unit at their position, and individuals played a fixed strategy of only producer or only scrounger in a tournament, whereas most experimental studies show that individuals use both tactics during a trial (e.g., [15], [16], [32], [33]). Here we incorporated travel times for scroungers and allowed individuals to play a mixed strategy by alternating between producer and scrounger within rounds. Importantly, we did not start with *a priori* differences between individuals by introducing fast and slow explorers in the model, or by assigning fixed foraging strategies (either producing, or scrounging) to individuals. Both the level of exploration and the scrounging probability were on a continuous scale (0–1) and were allowed to evolve (i.e., not fixed).

## The Model

### The Foraging Simulation

A population of size N_I_ individuals searched during a foraging round of T = 200 time units for N_P_ patches containing N_S_ indivisible food items. The patches were randomly distributed in an environment measuring 500 by 500 units (which can be assumed to be meters, though this can be scaled without loss of generality). The initial position of foragers in space was determined by choosing a random point in the environment (constrained to be outside of a margin of 10% of the total environment size from each side to avoid clustering at the edges; for a square environment of 500 units to a side, individuals were placed in the interior 450 by 450 unit square). At each time unit individuals could either be feeding or not. When individuals were feeding they would continue feeding in that patch, consuming one food item per time unit until the patch depleted. When individuals were not feeding, their action consisted of two steps. In the first step, they chose whether or not to move towards other flock members and in the second step they searched for food opportunities. Choosing to move towards flock members was selected according to the probability P_M_J_, the likelihood that an individual moves back to its conspecifics, calculated as:
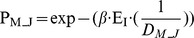
where E_I_ is exploration (ranging from 0 to 1), *β* is a scaling parameter that affects the rate of moving back to conspecifics and *D_M_J_* is the median distance between the focal individual and its conspecifics. The probability of moving back to conspecifics increased with decreasing exploration score, implying that slow exploring individuals move back to conspecifics from shorter distances as compared to fast exploring individuals. This is in agreement with the observation that slow exploring individuals show a lower tendency to split in smaller subgroups [22] and have a higher grouping tendency [18], [19], [34]. P_M_J_ increased with increasing median distance *D_M_J_*, ensuring the maintenance of group cohesion.

If an agent decided to move towards the other flock members, its direction of movement was calculated as follows: a new direction was chosen by averaging the directions from the focal individual to each conspecific, weighted by the distance to each conspecific so that closer conspecifics weighted the new direction more heavily, according to the weighting function:
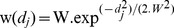
where w is the calculated weight as a function of distance, W is a weighting constant and *d_j_* is the Euclidean distance to a conspecific j. To handle situations in which every conspecific was too far away to affect the direction of the focal individual (all w(d_j_) close to 0), a new direction of movement would be calculated by adding a random component drawn from a Gaussian distribution (N(0,π/4)) to the previous direction (a correlated random walk). In the event that an individual reached the edge of the environment their direction was reversed (by adding or subtracting π rads = 180 degrees), with a small Gaussian random component from the same distribution (N(0, π/4)).

When searching for food an agent could either (1) search for food itself (play producer) with probability 1- P_S_I_ or (2) search for food discoveries of other individuals (play scrounger) with probability P_S_I_. (1) When producing, an individual investigated its close vicinity for food (as defined by a radius R_V_), and if food was encountered, it started feeding in the next time unit. If no food was found, it took a step randomly. Random movement was calculated by selecting from a uniform distribution over the set of new directions within 45 degrees on either side of the current direction. (2) When scrounging, an individual scanned the environment for producers exploiting a patch. The probability of detecting feeding producers P_F_J_ declined with distance *d_j_* to the forager: P_F_J_ = exp−(*d_j_*/H^2^). H determines the scrounging horizon; small values indicate that scroungers could only identify producers close by. If the focal individual identified a feeding producer (stopping at the first producer found), it moved in the direction of the discovered producer during the next time step with twice its normal step length. If the individual arrived in the next time step, it started feeding. If it did not arrive it continued in the direction of the foraging patch, provided that the patch still contained food items, until it reached the patch. If the patch was emptied during the movement, or if the scrounger did not find a forager in its initial search it moved randomly as described previously. A scrounger could only forage from the food discoveries of other foragers.

In all cases (moving to conspecifics or random movement), the length of the step S_I_ was a decreasing function of exploration: S_I_ = E_I_ * S_MAX_. S_MAX_ is the maximal step length. To avoid a potential value of zero, a small random Gaussian component (∼N(10,5)) constrained to be greater than zero was added to every individual’s step length. Step length increased with increasing exploration score, reflecting the observation that more explorative individuals are more often found in the leading edge of moving groups [20], [21], [35], [36].

The predation probability P_P_ represents the chance that an individual will be predated each round (from 0 to 80%); at the end of each time step a random uniform number was compared to the probability, and if it was lower, predation was applied to the population. To implement predation, the centroid (geometric center) of the population was first calculated as the average of each member’s position. Each member of the population then received a distance score from the centroid, c_i_ and an individual was chosen to be predated proportionally to its distance score (with individual probability 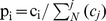); in essence, the individual farthest from the group center has the greatest chance of being predated and this chance increases as it is more isolated from other group members.

The maximum predation limit P_L_ was set to 5% of the population size (rounding half up); this meant that over the course of a single generation of the genetic algorithm, no more than 5% of the population would be lost to predation.

### The Genetic Algorithm

An individual’s strategy was encoded in a 2-locus real-valued chromosome, with locus 1 coding for the probability of scrounging P_S_I_ and locus 2 coding for the value of exploration E_I_. Both loci ranged from 0 to 1, and all individuals in a given population size N_I_ started the genetic algorithm with a randomly generated chromosome value chosen from a uniform distribution. At the end of each foraging round the number of consumed food items was computed for each individual and individuals were ranked on the basis of their fitness and the highest 60% were selected to reproduce; all other individuals were removed from the population, and selected individuals were chosen as parents in pairs with a probability proportional to their fitness (roulette-wheel) until the population was once again composed of N_I_ individuals. Crossover probability was 0.9 implying that the probability of a selected chromosome to remain unchanged in the next generation (apart from any changes due to mutation) is 0.1. Crossover was one-point linear crossover [37], [38]. One locus was chosen and the values from each parent at that locus were combined as follows: for parent alleles × and Y, offspring alleles X’ and Y’ were combined as X’ = Y+α(X−Y), Y’ =  × +α(X+Y), with α from U(0,1) and X’ and Y’ constrained to lie in {0,1}. The mutation rate was 0.1 (which is within the range of common usage for genetic algorithms [39]); if a locus was selected for mutation, it would be shifted from the old value by drawing a uniform random number between {−0.1,0.1} and adding that to the old value while constraining the values to lie within {0,1}. In the use of evolutionary computation techniques, it is important to check the resistance of the results to changes in parameter values such as mutation rate and crossover probability; to investigate this we conducted a sensitivity analysis for mutation (0.01–0.2), crossover (0–0.9), population size (50,100,500), and selection parameters (0.1–0.6) and found no evidence of changes in our results (data not shown). Our results were also robust to changes in foraging round duration and number of rounds (T and T_G_, data not shown). For the simulations reported here, we varied patch density, predation pressure and patch richness (see Table 1 for parameter ranges). All simulations were done in Python. For visualization purposes we have included a movie of the foraging simulations (Movie S1, Text S1).

**Table 1:** Parameters of the simulation (a) and behavioural variables (b).

Symbol	Meaning	Value or range
Parameters		
N_P_	Number of patches	5,10,20,30,40,50,60
N_F_	Number of indivisible food items in each patch	5,10,20,30,..,100
T	The duration of a round	200
T_G_	The number of rounds for each run of the geneticalgorithm	100
R_P_	Spatial radius of a foodpatch	10
R_V_	Radius of patch detectionfor producers	20
N_I_	Population size	50
P_L_	Predation limit	0.05
H	Scrounging horizon	10
B	Rate of moving back to conspecifics	25
P_P_	Predation probability	0,0.2,0.4,0.6,0.8
F_D_	Field dimension	500×500
Behavioural variables		
S_MAX_	Maximal step length	50
P_S_I_	Scrounging probability	0.0–1.0
E_I_	Exploration	0.0–1.0
P_F_J_	Probability of detecting producers	0.0–1.0
P_M_J_	Probability of moving back to conspecifics	0.0–1.0
W	Parameter of weighting function	50
S_I_	Step length	0.0–50.0

### The Evolution of Exploration and Scrounging

For each parameter combination (see Table 1), we ran one run of T_G_ = 100 rounds with five replicates per parameter combination for a total of 3850 runs. We analyzed the mean scrounging and exploration values, averaged over the last 10 rounds of each run to reduce the effects of stochasticity.

The convergence of the genetic algorithm was assessed by measuring fitness variability; in frequency-dependent selection, fitnesses of all members at equilibrium should be equal (accounting for the effects of discrete time and food intake). Therefore, we calculated and report herein the standard deviation of fitness values from the last 10 rounds.

## Results

**Figure 1:**
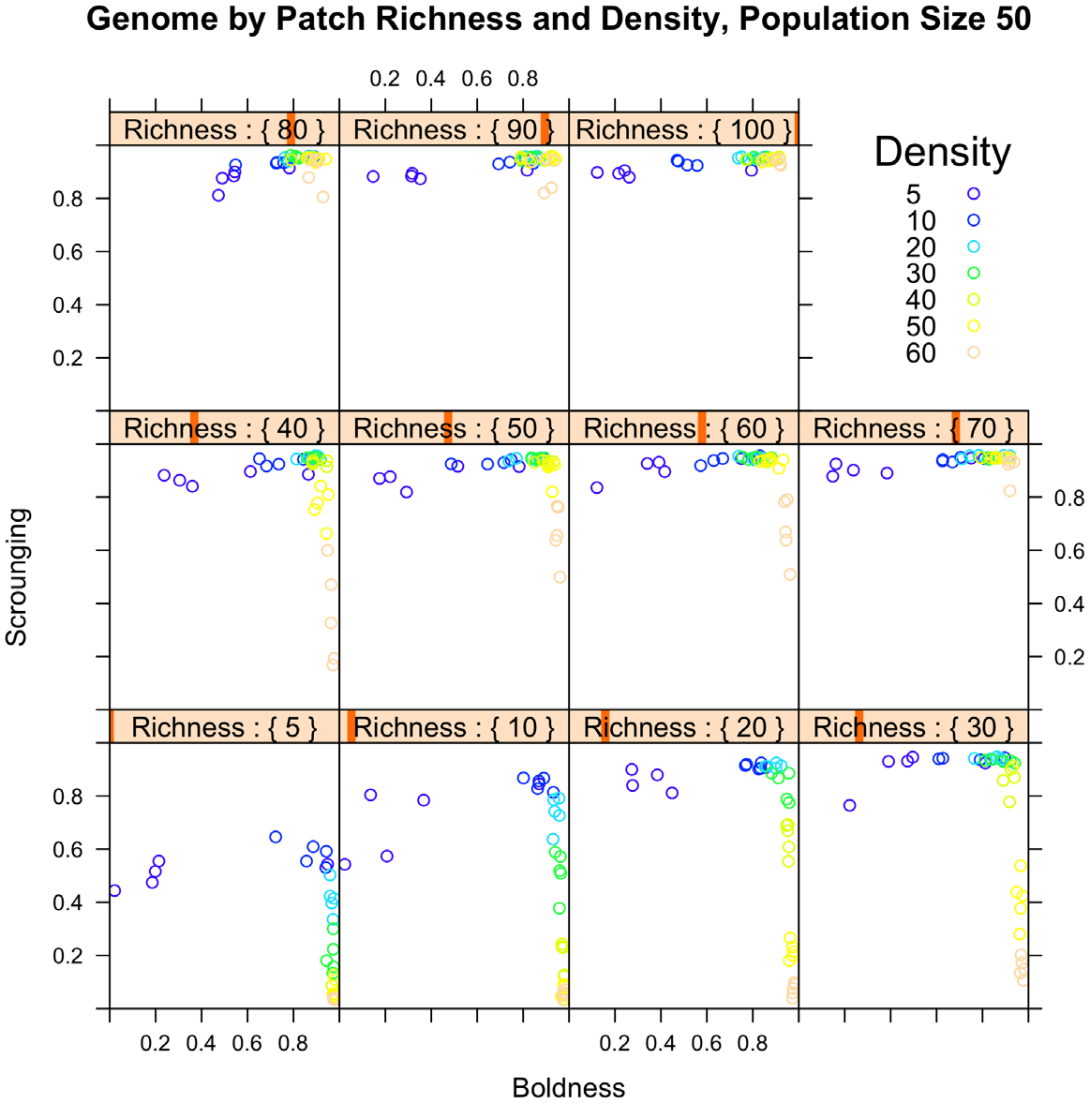
The effect of patch density and patch richness on exploration and scrounging values. An increase in patch density led to an increase in exploration and a reduction in scrounging, but the latter only under conditions of low/intermediate patch richness. An increase in patch richness resulted in increased scrounging, but there was no effect on exploration levels.

**Figure 2:**
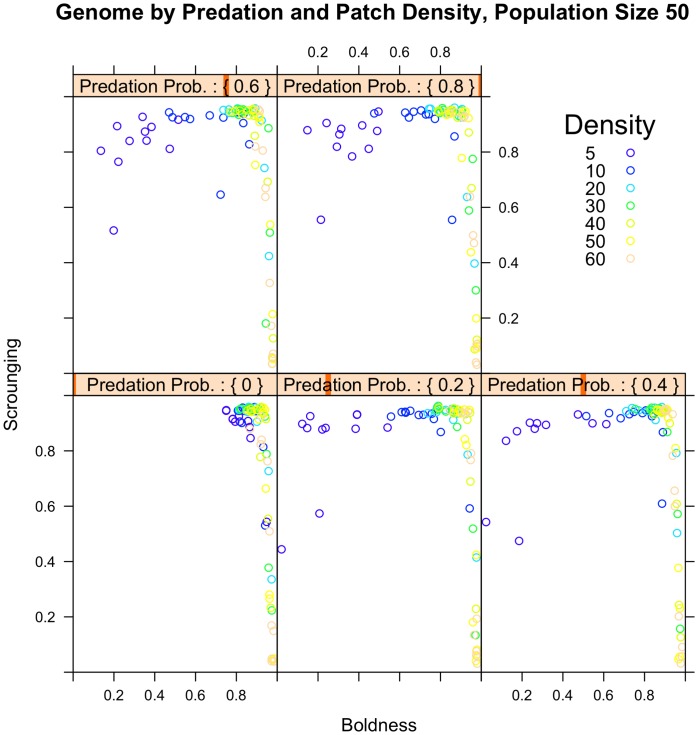
The effect of predation pressure and patch density on exploration and scrounging values. An increase in predation resulted in a reduction in exploration, but there was no effect on scrounging proportion.

An increase in patch density led to an increase in exploration: under conditions of low patch density slow exploring individuals were selected, but exploration increased rapidly with increasing patch density (Fig. 1). This pattern was consistent and appeared over the entire range of patch richness values (Fig. 1) and predation pressure, although the effect in the absence of predation was weaker (Fig. 2). An increase in patch density also led to a reduction in scrounging. This effect disappeared at high patch richness (Fig. 1). An increase in patch richness resulted in an increased scrounging, both for fast and slow explorers individuals (Fig. 1). There was, however, no effect of patch richness on exploration (Fig. 1).

In the absence of predation, only fast explorers emerged (Fig. 2). When predation was present, slow exploring individuals were selected and so exploration declined. There was no effect of predation on scrounging proportion (Fig. 2).

Exploration and scrounging became associated in the course of our runs: the slowest exploring individuals ending up with high scrounging propensities, whereas the fastest explorers had low scrounging probabilities (Figs. 1 and 2). High scrounging was observed over nearly the full range of exploration levels, except at the very extremes of exploration. Low scrounging was only observed at high values of exploration (Figs. 1 and 2). The GA showed low variability in fitness at the end of the run; the standard deviation in fitness values was less than 0.1 for 97% of the runs.

## Discussion

Our simulations demonstrated that individuals compromised between exploration and scrounging probability. Patch density and predation affected the evolution of exploration, whereas patch richness did not affect exploration. Patch richness and patch density affected the evolution of scrounging, whereas predation did not have an effect on scrounging. We discuss the effects of the three different environmental variables below.

The evolution of exploration was affected by patch density: increasing patch density led to an increase in exploration, whereas low patch densities select for increased levels of scrounging, leading to selection for slow exploring scroungers. Conditions of low patch density are known to favor scrounging [24], [40]. For scrounging to be profitable individuals need to remain close to each other, conditions that call for slow exploring individuals. As the number of patches increases patch discovery becomes more common and the equilibrium number of producers increase [41]. Producer success will be enhanced by the ability to distance themselves from conspecifics, which gives them more time to monopolize discoveries. These effects lead to selection for fast exploring producers at high levels of patch density and they suggest that gregariousness, the tendency to remain close to each other, will break down at high patch density by favoring spaced-out solitary foraging. Several models predict that group foraging is more likely to occur only when food patches are scarce and rich [42]–[44] exactly the situation where our simulation predicts the most scrounging and closest proximity and so the individuals with the lowest exploration scores.

Predation also affected exploration. As predicted by [45] we found that increasing the risk of predation reduced exploration. In our model the risk of being preyed upon increased with increasing distance from the flock center, meaning that fast exploring individuals moved further from the center of the group and suffered increased predation as a result. Previous work has suggested that differences in exploration are at least in part due to the consequence of differences in predation pressure [46]. It has even been suggested that the costs of being explorative are likely to disappear in the absence of predation [47]. In this scenario there is a trade-off between food intake rate and predation risk, with fast exploring individuals enjoying a higher food intake rate but also a higher risk of being predated. A positive correlation between exploration and food intake rate has indeed been reported in several species (for review see [48]) and there is now also evidence accumulating that fast exploring individuals suffer a higher predation risk [46], [49]–[51], due to an increased tendency to expose themselves to risky situations. A decrease in exploration with increasing predation only occurred at low patch densities (Fig. 2). When patch density was high, exploration levels were also high across all predation pressures. We suggest that at high patch density it still pays off to be explorative even under high predation pressure because the benefit to monopolizing a patch outweighs the chance of predation. At low patch densities, exploring does not pay off equally since returns of exploring are low as it is difficult to find a food patch. Additionally, at high patch density, explorers can find patches less far from the center of the group. Since predation risk increases with increasing distance from the center it is likely that explorers suffer less predation because they remain closer to the group center when the patch density is high.

Patch richness affected scrounging probability: increasing patch richness, favored high conditions of scrounging. These findings fit well with both empirical and theoretical results for producer – scrounger games [3], [24], [52]–[54], and show that also in a spatially explicit producer-scrounger game environments with high patch richness favor the evolution of scrounging (see also [24]). Patch richness did not affect exploration, in contrast to patch density and predation which both affected the evolution of exploration. We suggest that this is because patch richness is not a spatial variable, unlike patch density and predation which are both spatial variables. One of the mechanisms that may play a role in linking exploration and scrounging are the spatial dynamics of individuals. It has been hypothesized that social information use (scrounging) should be more profitable for slow explorers that stay closer to conspecifics than fast explorers [16], [55], [56] because the value of information is expected to decrease with increasing distance [57]. For example, in a producer-scrounger game the success of playing a producer depends on the producers’ ability to distance themselves from conspecifics, whereas the success of playing scrounger depends on the scroungers’ ability to be at close proximity to producers [23], [25]. We believe that spatial dynamics are the single most important reason for the various effects we found on exploration. We verified the relationship between exploration score and distance from the population centroid, confirming that distance from the population centroid increased with increasing exploration score (E_I_) (data not shown).

In our simulation a tendency to increase one’s distance from conspecifics is achieved by increasing exploration, meaning that slow explorers forage at close proximity of each other, whereas fast explorers are more spaced out. Thus, the spatial arrangement of food resources can affect the optimal social attraction rules between agents (i.e., exploration), the optimal level of scrounging and the interaction between both. In Scottish blackface sheep, *Ovis aries*, the spatial distribution of fast and slow explorers differed with fast explorers splitting into subgroups at smaller group sizes than slow explorers [22]. The differences in spatial distribution could be simulated by a model that included simple rules on sensitivity to crowding and social attraction [58]. Although our model is different as it runs over an evolutionary time scale both our model and theirs show the importance of differences in social attraction rules on the spatial dynamics of individuals foraging in groups. Clearly, how exploration affects social attraction rules between group living individuals and how this in turns affects collective processes is an exciting avenue for further research.

We also investigated variation between individuals within runs (i.e., within a given environment) by investigating whether there was a polymorphism in scrounging and exploration (Text S2). Within each run populations evolved towards monomorphism where a single optimal level of exploration characterized all the individuals in the population (Text S2). We found thus no evidence for a stable co-existence of different (personality) types within one population submitted to a given assortment of environmental conditions. However, across runs there was a general association between exploration and scrounging probability: high rates of scrounging were observed over nearly the full spectrum of exploration values, whereas high rates of producing were only observed at high exploration values. This conforms to experimental evidence in barnacle geese, *Branta leucopsis*, that fast exploring geese produced patches faster than slow explorers, whereas exploration did not affect the rate of joining patches [59]. Our predictions also confirms the observation that fast exploring individuals of several different species approach food patches (‘producing’) quicker than slower explorers [13], [19]–[21], [36] but see [14]. Our results anticipate that cases of slow explorers that produce should be rare, whereas fast explorers are expected to act both as producers and as scroungers. Though we did not find evidence for a stable co-existence of different personality types, our simulations do show that differences in patch density and predation pressure result in a broad range of different optimal levels of exploration across runs. Differences in selection pressures (either in space or time) in a social foraging game may generate different optimal exploration levels, suggesting that spatio-temporal dynamics (i.e., fluctuating environments) may cause variation in exploration levels between populations [26], [60], [61] or between individuals within populations when individuals use different micro habitats due to habitat specialization as reported in numerous species [62]–[64].

To conclude, we have shown that individuals compromised between exploration and scrounging probability and that this depended on the environment: under conditions of low patch densities slow exploring scroungers were favored whereas high patch density favored fast exploring individuals that favored producing (at low patch richness) or scrounging (at high patch richness). In high predation environments slow exploring individuals were selected for but only at low to intermediate patch densities. We did not find a divergence of exploration ‘types’ within runs but there was a general association between exploration and scrounging when comparing the outcomes across different environments: high rates of scrounging were observed over nearly the full spectrum of exploration values, whereas high rates of producing were only observed at high exploration values. Our results indicate that the spatial arrangement of food resources can affect the optimal social attraction rules between agents, the optimality of foraging tactic and the interaction between both. This can explain differences in exploration and scrounging between individuals of different environments or within environments when individuals use different microhabitats.

## Supporting Information

Movie S1 **Visualization of five runs of the genetic algorithm showing producers and scroungers in the foraging environment.**
(MOV)

Text S1 **Legend for Movie S1.**
(DOCX)

Text S2 **Results of model-based cluster analysis investigating polymorphism in scrounging and boldness.**
(DOCX)
