# Supplementary material for: The Effect of Exploration on the Use of Producer-Scrounger Tactics
Source: PLoS One. 2012 Nov 21;7(11):e49400. doi: 10.1371/journal.pone.0049400 (PMC3503990; doi:10.1371/journal.pone.0049400)
Supplement: Text S1 — Legend for Movie S1. (DOCX) [file pone.0049400.s002.docx]

**Text S1 - Legend Movie S1**

A partial run of the genetic algorithm. In the leftmost window is a visualization of the foraging simulation; green circles are food patches, white dots are individuals using the producer tactic, blue dots are individuals using the scrounger tactic, and the red dot is a focal individual. Arrows are directed vectors, the direction of the individual’s movement in the current time step. The next four windows show simulation and genetic algorithm plots. Clockwise from top-left: average foraging success by time in the current foraging round, mean population chromosome values by locus (scrounging and boldness), chromosome scatterplot showing individual chromosome values in two-dimensional trait space, and fitness values over rounds (population mean, best of generation, and mean producer or scrounger fitness). The final windows are for output, diagnostic, and control purposes, respectively. Pictured are the first 20 rounds of a run with parameters: T = 200, T_G_ = 100, N_P_ = 100, P_P_ = 0.05.
