# Supplementary material for: The Effect of Exploration on the Use of Producer-Scrounger Tactics
Source: PLoS One. 2012 Nov 21;7(11):e49400. doi: 10.1371/journal.pone.0049400 (PMC3503990; doi:10.1371/journal.pone.0049400)
Supplement: Text S2 — Results of model-based cluster analysis investigating polymorphism in scrounging and boldness. (DOCX) [file pone.0049400.s003.docx]

**Text S2**

**Methods**

For each run of the genetic algorithm, we searched for a polymorphism in scrounging and boldness using model-based cluster analysis on the final scrounging and boldness values for each member of the population using the Mclust package in R [1,2]; Mclust provides the optimal model according to BIC (Bayesian Information Criterion) for expectation maximization in Gaussian mixture models. If a clear polymorphism of boldness and scrounging values was present (e.g., bold producers and shy scroungers), the cluster analysis would be expected to select a model with two clusters.

**Results**

There was no evidence for a dimorphism in boldness. Model-based cluster analysis provided no preference for 2 cluster model (Table 1), and inspection of the data revealed that this was due to low variance in both scrounging and boldness across runs (mean σ_scr_ = 0.049, mean σ_bold_ = 0.038); the results of cluster analysis are bound to be unstable when overall variance is so restricted. Every run converged to a single value of boldness (max σ_bold_ = 0.067), and only 54 runs failed to converge to a single value of scrounging (σ_scr_ > 0.1). Inspection of these runs confirmed the results of the statistical analysis in finding that no dimorphism in either boldness or scrounging existed.

**References**

Fraley C, Raftery AE (2002) Model-based clustering, discriminant analysis, and density estimation. J Am Stat Assoc 97: 611-631.

Fraley C, Raftery AE (2006) MCLUST Version 3 for R: Normal mixture modeling and model-based clustering. Technical Report No. 504, Department of Statistics, University of Washington.

**Table 1**

Optimal number of components selected in a cluster analysis of each run of the genetic algorithm.

| Model components | 1 | 2 | 3 | 4 | 5 | 6 | 7 | 8 | 9 |
| --- | --- | --- | --- | --- | --- | --- | --- | --- | --- |
| Number of runs | 116 | 394 | 507 | 534 | 548 | 448 | 509 | 398 | 396 |
